# Characterization and Functions of Protease-Activated Receptor 2 in Obesity, Diabetes, and Metabolic Syndrome: A Systematic Review

**DOI:** 10.1155/2016/3130496

**Published:** 2016-02-23

**Authors:** Satomi Kagota, Kana Maruyama, John J. McGuire

**Affiliations:** ^1^Department of Pharmacology, School of Pharmacy and Pharmaceutical Sciences, Mukogawa Women's University, Nishinomiya, Hyogo 6638179, Japan; ^2^Cardiovascular Research Group, Division of BioMedical Sciences, Faculty of Medicine, Memorial University, St. John's, Newfoundland and Labrador, Canada A1B 3V6

## Abstract

Proteinase-activated receptor 2 (PAR2) is a cell surface receptor activated by serine proteinases or specific synthetic compounds. Interest in PAR2 as a pharmaceutical target for various diseases is increasing. Here we asked two questions relevant to endothelial dysfunction and diabetes: How is PAR2 function affected in blood vessels? What role does PAR2 have in promoting obesity, diabetes, and/or metabolic syndrome, specifically via the endothelium and adipose tissues? We conducted a systematic review of the published literature in PubMed and Scopus (July 2015; search terms: par2, par-2, f2lr1, adipose, obesity, diabetes, and metabolic syndrome). Seven studies focused on PAR2 and vascular function. The obesity, diabetes, or metabolic syndrome animal models differed amongst studies, but each reported that PAR2-mediated vasodilator actions were preserved in the face of endothelial dysfunction. The remaining studies focused on nonvascular functions and provided evidence supporting the concept that PAR2 activation promoted obesity. Key studies showed that PAR2 activation regulated cellular metabolism, and PAR2 antagonists inhibited adipose gain and metabolic dysfunction in rats. We conclude that PAR2 antagonists for treatment of obesity indeed show early promise as a therapeutic strategy; however, endothelial-specific PAR2 functions, which may offset mechanisms that produce vascular dysfunction in diabetes, warrant additional study.

## 1. Introduction

Obesity, diabetes, and metabolic syndrome are risk factors for cardiovascular disease. Insulin resistance and high blood glucose levels can lead to endothelial dysfunction, a cardiovascular complication of these dysmetabolism states and a common pathology of cardiovascular disease [1]. Endothelial dysfunction impairs regulation of vascular smooth muscle tone and vasodilation, which reduces oxygen supply and inhibits the capacity of tissues and organs to meet changes in metabolic demand [2]. Improving cellular metabolism and preserving, restoring, and/or rescuing endothelial cell-regulated vascular functions like vasodilation are desirable features for new therapeutics.

**Figure 1:**
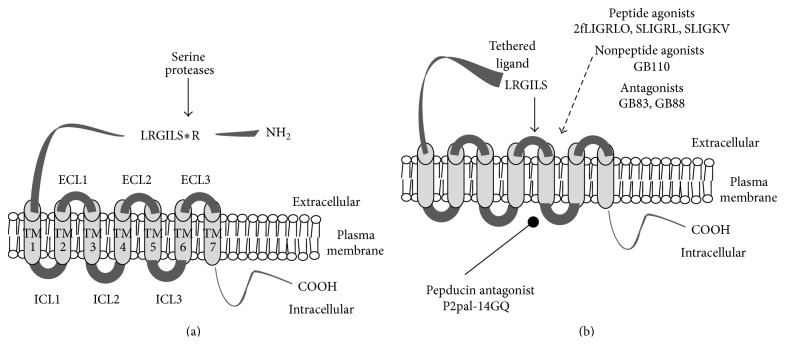
Activation of protease-activated receptor 2. (a) PAR2 is a seven-transmembrane domain cell surface receptor that can be activated by serine proteases which recognize a substrate sequence on the N-terminus (-NH_2_) located in the extracellular space. To highlight the unique mechanism of action a simplified cartoon shows the arrangement of the nonactivated PAR2 protein sequence (ribbon) in a cell plasma membrane. Asterisk indicates the site of proteolytic cleavage of mouse and rat PAR2 associated with serine proteases, including trypsin, human mast cell *β*-tryptase, matriptase, and several human kallikreins. (b) Following proteolytic cleavage, the newly revealed N-terminus (shown as  LRGILS) acts as a tethered ligand that interacts (solid arrow) with the extracellular loop-2 (ECL-2) domain and induces the activated state of the receptor. Alternatively, receptor activating peptides (2fLIGRLO, SLIGRL, and SLIGKV), and nonpeptide agonists (GB110) can activate PAR2 without the participation of proteases (dashed arrow). Also, shown are the proposed sites of action of different classes of PAR2 antagonists, that is, GB83, GB88 (dashed arrow) and PAR2 pepducin P2pal-14GQ (oval arrow). Peptide sequences are identified by their amino acid sequences using the standard capitalized one-letter abbreviations. All PAR2 activating peptides were synthesized as amides. Sequence starting with 2f indicates N-terminal modification with a 2-furoyl functional group. COOH: carboxy terminus; ECL: extracellular loops domains 1 to 3; ICL: intracellular loop domains 1 to 3; TM: transmembrane loop domains 1 to 7.

This study is a systematic review of the literature providing evidence that proteinase-activated receptor 2 (PAR2) is involved in obesity, diabetes, and metabolic syndrome. PAR2 is a cell surface receptor that is activated by endogenous serine proteinases or pharmacologically by synthetic ligands (Figure 1) [3, 4]. On the one hand, PAR2 activation could preserve blood flow associated with specific endothelial cell mechanisms; on the other hand, PAR2 activation could also stimulate inflammation pathways, which may impair cellular metabolism, produce insulin resistance, and promote obesity and diabetes [5]. Our objective for this review was to gain a better understanding about PAR2 effects—especially its activation versus inhibition—in studies of obesity, diabetes, and metabolic syndrome. Two specific questions were asked: How is PAR2 function affected in blood vessels? What role does PAR2 have in promoting obesity, diabetes, and/or metabolic syndrome, specifically via the endothelium and adipose tissues? This review identifies current trends and knowledge gaps about PAR2 actions in obesity, diabetes, and metabolic syndrome. Addressing these gaps may improve the strategies to address obesity and/or diabetes or raise important issues to be addressed as pharmaceutical development proceeds with PAR2-based drugs.

In the vasculature, PAR2, a seven-transmembrane class-A GPCR [6], is constitutively expressed in endothelial cell [7] and inducible in endothelial cell and vascular smooth muscle cells [8, 9]. The canonical description for the mechanism of PAR2 action is that specific PAR2-activating compounds and serine proteinases activate Gq-alpha, which via phospholipase C activity increases intracellular IP3 and activates IP3R and results in the release of internal stores Ca^2+^ as shown in [10]. PAR2 signal transduction mentioned above is recognized as being more complex (reviewed in [11]) than a single agonist-receptor pathway. Thus, PAR2 signalling by the internalized receptor [12], transactivation of other receptors [13], subcellular localized receptor distributions [14], tethered-ligand disarming [15], and agonist-biased signals [16] contribute to a complex system of PAR2 mediated cell responses.

Serine proteases like trypsin [4, 17] and human mast cell *β*-tryptase [18], matriptase [19], and several isoforms of human kallikreins [20] activate PAR2 by a mechanism involving a self-contained tethered ligand (Figure 1) [6], which is produced by proteolytic cleavage within a specific sequence of PAR2's amino terminus and binds to sites in its extracellular loop 2 domain [21]. Nonserine proteases, including elastase and cathepsin S, have also been reported to recognize substrate sequences within the N-terminus of PAR2 and thus produce alternate truncated extracellular N-terminus [15, 22]. Some of these truncations will effectively disarm the receptor by removing the region used for activation by other proteases and other truncations may induce an activation of PAR2 signal transduction [23].

A broad range of pharmacological tools (Figure 1) are available for examining PAR2 (reviewed in [24]), including PAR2-activating peptides [25–28], small molecule PAR2 agonists [29–32] and antagonists [33, 34], cell permeable pepducins [35], and anti-receptor antibodies [36]. PAR2-activating peptides are short peptides synthesized to match the N-terminal amino acid sequence of the PAR2 tethered ligand revealed by trypsin and trypsin-like serine proteases [27]. For example, SLIGKV and SLIGRL match the N-terminus sequences of human and rodent (mouse and rat) PAR2, respectively [26]. Modifications (e.g., substitutions and additions) to the synthetic PAR2-activating peptides change the potency, increase in vitro resistance to peptidases, and provide sites to tag with labels for ligand-binding [37] and receptor visualization studies [38]. 2-Furoyl-LIGRLO (2fLIGRLO) is one example of a compound derived from the template SLIGRL and is the most potent PAR2 peptide agonist in use since its discovery [28]. GB110 is a nonpeptide compound with good potency and selectivity for activating PAR2 [29, 33]. Researchers examined the effect of changing the carboxy-terminus of GB110 to different spiropiperidines and as a result identified two compounds, GB83 and GB88, which are selective and potent inhibitors of PAR2-activation by either activating peptides or serine proteases [33]. This distinction was particularly notable because another PAR2 antagonist, ENMD-1068, inhibited only enzyme activation of PAR2 [39]. GB88 administered in vivo reduced inflammation in various models of inflammation [40, 41]. However, the more recent study provides evidence that GB88 is a selective antagonist of PAR2-mediated intracellular calcium-release and a partial agonist of other PAR2-mediated signal transduction, for example, ERK phosphorylation [41]. A separate group of researchers has developed a different PAR2 antagonist, C391, which blocked multiple PAR2-mediated cell signaling pathways [34]. A different technology strategy for pharmacological inhibition of PAR2 is the development of pepducins, which are a class of lipid soluble peptide compounds that target the intracellular regions of GPCR [42]. PAR2 pepducins (e.g., P2pal-21F and P2pal-18S) are amino-terminus palmitoylated peptides that use the intracellular loop three of PAR2 as a template amino acid sequence [35]. P2pal-21F is a nonselective agonist of PAR2 and PAR1 whereas P2pal-14GQ is a selective antagonist of PAR2 [35]. P2pal-14GQ is the most potent of a series of PAR2 pepducins that inhibited PAR2-mediated cell signaling and was anti-inflammatory in a PAR2-depenent inflammation model [35].

In endothelial cells, PAR2-mediated Ca^2+^-release activates nitric oxide synthase [25] and Ca^2+^-activated potassium channels [43], relaxes vascular smooth muscle, dilates arteries, increases tissue perfusion, and lowers blood pressures [44]. Yet in contrast to these potentially beneficial effects on cardiovascular function, studies have provided evidence demonstrating PAR2 activation can promote inflammation responses, for example, by inducing cytokine release [19] and adhesion molecule expression [45]. However, PAR2 mediated pathophysiology is not associated with any singular serine proteinase, which would appear to leave a wide scope of potential roles for the receptor and potential mediators in obesity, diabetes, and metabolic syndrome.

## 2. Methods

Studies were selected for review by searching PubMed and Scopus databases (last search updated July 2015). In one arm of the search, the terms were ((par2 or par-2 or f2rl1) endothelium) and (diabetes or obesity or adipose or metabolic syndrome). PAR2 and PAR-2 have been more commonly used than f2rl1 (factor 2 receptor like 1), which is the most recent name assigned to the gene encoding PAR2 and highlights the gene homology shared with factor 2 receptor (f2r1, PAR1, or the thrombin receptor). In the other arm of the search, the keywords were ((par2 or par-2 or f2rl1) and (diabetes or obesity or adipose or metabolic syndrome) not endothelium. These searches produced 10 and 35 results. Three additional studies were identified by citations in the search articles. Each study was abstracted and reviewed for relevance and, subsequently, content. Tables 1 and 2 list all the included studies from first [46–48, 50–53] and second arm [54–60, 62–67], respectively. Reviews, editorial articles, and papers that did not pertain to protease-activated receptors or deemed to have peripheral or limited direct relevance to diabetes, obesity, or metabolic syndrome were excluded from further study.

## 3. Results

### 3.1. PAR2 Endothelial Cell Mechanisms in Obesity, Diabetes, and Metabolic Syndrome

PAR2 expression on endothelial cell [7] and adipocytes [48] is constitutive. Inflammatory mediators [68] and dietary fatty acids [63] can induce PAR2 expression in endothelial cells and adipocytes, respectively. From separate studies, it is known that PAR2 activation by specific activating compounds and enzymes cause endothelial cell-mediated vascular smooth muscle relaxation, dilate blood vessels, increase blood flow, and lower blood pressures [44].

All studies listed in Table 1, except for a single study [48] reported PAR2-mediated relaxations of blood vessels that were preserved or increased in animals with obesity [53], diabetes (type 1 [46] or type 2 [47, 50, 51]), or metabolic syndrome [52]. These results on their own suggest that PAR2 activation may preserve local or regional blood flows in the face of endothelial dysfunction. The shared result is interesting because several different rodent models associating endothelial dysfunction with metabolic status were utilized. In these studies, PAR2 was compared with muscarinic [46, 47, 50–52] or bradykinin [53] receptor mechanisms of vasodilation; both of which were impaired, consistent with their use as internal references for endothelial dysfunction in these models. Unfortunately, Li et al. [48] did not provide data to allow comparison of PAR2 endothelial cell-mediated vasodilation between healthy (C57B6) and noninsulin dependent diabetes models (type 2; TallyHo mice). The cellular (endothelial cell versus vascular smooth muscle cell) and subcellular mechanisms explaining the PAR2 mediated relaxations vary—for example, nitric oxide [47, 50, 52], cyclooxygenases [46, 50] or Ca^2+^-activated K^+^ channels [51, 53] have been inferred as primary mediators—between studies. Differences in the choices of experimental models and types of blood vessel preparations (aorta [46, 47], coronary arterioles [50], small mesenteric artery branches [51, 52], middle cerebral artery [53], and superior-mesenteric artery [52]) may contribute to some of the mechanistic variations between each study.

In the studies listed in Table 1, all of the experimental group animals displayed elevated blood glucose [47, 48, 50, 51, 53] and or urine glucose [46, 51, 52] levels, consistent with the premise of metabolic dysregulation. In two of the four studies with type 2 diabetes models, the diabetic animals (db/db mice) were obese and weighed more than lean controls [50, 51]. Glucose intolerance, high levels of serum insulin and blood lipids, larger body masses, and high systolic arterial blood pressures were confirmed in the rat model of metabolic syndrome [52]. Underlying causes of hyperglycemia, insulin resistance, and/or obesity included genetic propensities for type 1 [46] and type 2 diabetes [47, 48, 50–52] and a high-fat high-carbohydrate diet [53]. As shown in Table 1, the metabolic phenotypes of the experimental groups were broadly categorized: nonobese diabetic [46, 47], obese diabetic [48, 50, 51], diet-induced obesity [53], and metabolic syndrome (high blood pressure [52]). One study [52] examined the reversal of hypertension and elements of endothelial dysfunction on PAR2 and blood vessel function after treatment of animals with telmisartan, an angiotensin-II type 1 receptor antagonist. Two studies by independent research groups [50, 51] examined obese diabetic db/db mice. However, each group looked at outcomes in different vasculatures. Treatment in vivo with a putative PAR2 antagonist peptide reportedly reversed endothelial dysfunction in the coronary vasculature of the db/db [50]. Each of the other studies in Table 1 used a different experimental model; some models produced similar metabolic phenotypes despite being based in different species or strains of rodents.

Only two studies reported using nonobese diabetic animals to study PAR2 in the vasculature. These studies found the same trend for sustained PAR2 vasodilation [46, 47]. In nonobese type 1 diabetic female mice [46], PAR2 mediated dilations of aortas were preserved by an increased release of COX-2 dependent prostaglandins. By comparison with another nonobese rodent model of type 2 diabetes, male Goto-Kakizaki rats [47], a cyclooxygenase inhibitor had no effect on PAR2 mediated relaxation of the superior mesenteric artery; the proposed explanation was an increase in PAR2 expression that compensated for decreased vasodilator mediators. No direct comparisons between obese and nonobese diabetic models have been reported.

Age-dependent outcomes were assessed in a few studies as a part of the model employed [46, 52, 53]. In such cases, time-course changes in the sensitivity to PAR2 appeared to increase with the progression of the metabolic disease [46, 52, 53]. Within the studies listed in Table 1, the effects of sex on PAR2-dependent responses in blood vessels have not been examined; most of the studies exclusively used male animals [47, 51–53]. Indeed the few studies that included female subjects did not account for sex in reporting or analyzing data [46, 48, 50]. All studies in Table 1 were conducted in rodents. Mouse strains included CD-1, FVB/N, and NOD/Ltj [46]; TallyHo [48]; C57BL6 [48, 51]; rat strains included Wistar [47, 52] and Sprague-Dawley [53]. Organ bath [47, 52] and small wire-myograph [46, 48, 51] bioassay methods outnumber the isolated perfused vessel preparations [50, 53] for assessing endothelial function in blood vessels. Endothelial function was measured in all cases by the reversal of blood vessel constriction, using pretreatment with agonist that varied in each preparation. PAR2-activting peptides, SLIGRL [46–48, 53] or 2fLIGRLO [48, 50–52], were used as agonists for PAR2 activation. Only one study [51] also used trypsin, a serine protease that activates PAR2 at low concentrations and PAR1 at high concentrations. Similarly only Li et al. [48] examined blood vessel function with PAR2 in the presence of perivascular adipose tissue, identifying adipose-derived relaxing factors elicited by PAR2 agonist (SLIGRL). Interestingly, the PAR2-inactive peptides (LRGILS and 2fOLRGIL) caused a PAR2-indepdent release of a pharmacologically distinct adipose-derived relaxing factor [48].

In a couple of studies [46, 50], immunohistochemistry data provided preliminary qualitative evidence corroborating the increased expression of PAR2 within the selected blood vessel. Quantifying PAR2 expression in tissues has been problematic because the same tools (antibodies) used in Western blot methods demonstrated evidence of off-target signals [51, 69] and, thus, challenge the validity of concluding that PAR2 expression increased [46, 47, 50]. Quantitative real-time PCR has been used to assess* par2* gene expression [51, 52] and is indirect evidence of protein expression. In general, evidence of the subcellular distribution of PAR2 within endothelial cells of the vessels is lacking in these studies, but based on functional studies (i.e., removing the endothelium and using genetic PAR2 knockouts) the expression of PAR2 in endothelial cells is critical to the blood vessel function in all except two studies [46, 48]. However, between these latter studies, only Roviezzo et al. [46] compared endothelial cell-mediated vasodilation by PAR2 between the healthy and disease states. Previously, the other investigators provided evidence of endothelial dysfunction in aortas of TallyHo mice, based on experiments using only acetylcholine as the primary agonist [49].

Metabolic syndrome was examined in a single experimental model [52] that combined high arterial blood pressure with the altered metabolic parameters. This SHRSP.ZF rat model points to sustained nitric oxide-mediate mechanisms underlying PAR2 activation of arteries [52]. Interestingly, angiotensin-II receptor 1-antagonist treatment in this same model did not affect the sustained PAR2 mechanism and restored function to other endothelial cell agonists by reestablishing nitric oxide-mediated vasodilation [52]. A number of factors in this model, including age, sex, and disease progression, warrant further study to delineate the regulation of PAR2 under the conditions of metabolic syndrome. This model [52] in particular may be useful for following up the cardiometabolic consequences of PAR2 function inferred by the studies in Table 2.

### 3.2. PAR2 Signalling Mechanisms in Obesity, Diabetes, and Metabolic Syndrome

#### 3.2.1. Experimental Models

As summarized in Table 2, researchers have applied separately in vitro and in vivo methodologies for examining the role of PAR2 signalling in obesity and diabetes, outside of the context of endothelium function [54–60, 62–67]. Overall, there is a trend of finding increased PAR2 expression in tissues from obese humans [63] and rodents, which included obesity with or without diabetes. Causes of obesity and diabetes in these models included genetic mutations [56, 58, 59], high fat diets [57, 67], or T cell mediated pancreatic islet destruction [55]. In some but not all studies, the increases in mRNA expression or immunospecific staining of tissues were corroborated by functional responses to agonists of PAR2 [54–56, 60, 63]. PAR2-activating peptides (2fLIGRLO [56, 63], SLIGRL [54, 55, 63], and SLIGKV [64, 65]) were more commonly used as agonists, with fewer studies having used trypsin activation of PAR2 [54, 60, 64]. Interesting trends in the research themes include studies of transactivation of PAR2 by cell specific receptors, for example, the cytoplasmic tail of tissue factor [57, 67] or epidermal growth factor receptor, and the regulation of metabolic pathways by PAR2, for example, AMP-activated kinase [56, 57, 67]. Such research seeks answers to address the question of how PAR2 is linked to regulation of cell metabolism and proposes novel interactions between metabolism and the immune system.

There is an increasing trend in the field of PAR research towards identifying novel activation pathways by endogenous proteases or nonserine proteases [58, 64, 65]. Studies listed in Table 2 have provided relatively weak evidence for any relationship between a specific protease [58, 64, 65] as being an endogenous mediator of PAR2's role in diabetes and obesity. As we found with the studies of PAR2 with the vasculature, the studies [54–60, 62–67] listed in Table 2 have not systematically investigated age- or sex-related differences.

#### 3.2.2. Signalling Pathways Differences/Heterogeneity

As shown by examples cited in this review, PAR2 intracellular signalling pathways vary by cell type and experimental conditions. Indeed the selectivity of activators for PAR2 Ca^2+^-dependent versus Ca^2+^-independent signaling has been proposed as an explanation differentiating actions of PAR2 by different enzyme agonists [70]. Recent studies indicate distinct roles for regulation of gene expression by PAR2 based on whether it is localized at the plasma membrane versus the cell nucleus [14, 71]. Thus, recognition of PAR2 agonist-biased signalling is warranted for future studies that examine this receptor in disease models and consider the therapeutic potential of various PAR2 modulating compounds.

Blocking PAR2 signalling with small molecule antagonists and antibodies has been tested and resulted in reducing or normalizing adiposity and some metabolic parameters in the obesity models [57, 63, 67]. In one case using the diet-induced obesity/metabolic syndrome rat model [63], supplementary data provided evidence that GB88, a biased PAR2 antagonist, treatment blunted the effect of HCHF-feeding on several indices of cardiac function and the interstitial collagen content in the left ventricular myocardium; however, cardiac output was not different between controls and treatments. Systolic blood pressures of HCHF + GB88 group were less (~10%) than HCHF alone. Further examination of GB88 action in vivo when placed in combination with selective PAR2 antagonists, for examples, pepducin P2pal-14GQ [35], could provide further insight into cellular mechanisms. Studies in* par2* knockout mice indicated eliminating* par2* function had a modest effect on raising systolic blood pressures [72] and, thus, may counter the benefits of lowering the rate of adiposity gain under a high fat diet. Integrating the models of PAR2 function in the vasculature with its concomitant actions elsewhere, which promote obesity, is a logical step toward a better understanding of the balance of PAR2 therapeutics for metabolic dysfunction and cardiovascular risk.

## 4. Discussion

We conducted this review in order to evaluate the role of PAR2 in obesity, diabetes, and metabolic syndrome as well as identifying trends in this research field. We found that PAR2 was linked to obesity, diabetes, and metabolic syndrome by two separate, but not mutually exclusive research themes, which we identified by their focus on PAR2-mediated endothelial cell functions or not. This division of the literature along two themes is relevant because endothelial dysfunction is a characteristic factor of cardiovascular complications in diabetes. Strategies for pharmaceutical interventions targeting PAR2 should account for both vascular function and metabolism effects in diabetes.

The first theme we identified in the literature centers on the role of PAR2 on endothelial cells and their function or dysfunction in regulation of circulatory control in metabolic syndrome obesity and or diabetes [46–48, 50–53]. In these studies, a case is made for the presence of sustained PAR2 mediated vasodilation in the diabetic, obese, or metabolic syndrome state that is consistent with the broader literature about preserved PAR2 functions on arterial vasculature, for example, in other models of circulatory diseases, conditions (ischemia), and injury [45, 51, 73–79]. Under this theme, studies focused on the functional local consequences of PAR2 activation on blood vessels and strategies have applied various rodent models [46–48, 50–53]. Tissue responses to PAR2 activation are assayed by the endothelium dependent or independent mechanism in the functional responses of blood vessels [46–48, 50–53]. These studies vary by the age of animals, sex, species, and the vessel types (Table 1). In some cases the mechanisms are explored and the mediators of the pathways include nitric oxide, cyclooxygenases, and endothelium-dependent hyperpolarization [46–48, 50–53]. A development in the literature is a role proposed for PAR2 as a regulator of vascular tone via perivascular adipose tissue, specifically via adipocytes [48]. Others reported finding PAR2 expression in a nonadipocyte fraction of PVAT, which included infiltrating immune cells [63]. PAR2 differentiates from other GPCR by its activation of paracrine factors from perivascular adipose tissue that affect function of the blood vessels [48]. Such studies bridge a blood vessel-centered focus on circulatory function with evidence that PAR2 plays a role in inflammation pathways that link to metabolic dysfunction, outlined in the next section.

In the second theme, researchers have focused on models residing primarily outside of the endothelial cell domain [54–60, 62–67]. These studies have provided insight into detailed intracellular signalling mechanisms that build a case for PAR2 promotion of obesity. A developing trend in this theme is a divergence on the focus of PAR2 regulation mediated solely by pairing of GPCR with an agonist; rather studies present PAR2 as a coreceptor associated with the complex signaling by other receptors, for example, by the cytoplasmic domain of tissue factor [57, 67]. Along a similar line, investigators are examining PAR2 modulation of cell functions mediated via *β*-arrestin dependent mechanisms [56]. Converging evidence points to a common pathway of AMPK regulation by PAR2 that can be modulated by *β*2-arrestin expression [56, 57, 67]. Demonstrating PAR2 is a modulator AMPK, a key regulator of cell metabolism, is a significant advancement in understanding its effects on energy regulation in the whole organism and supports the notion of interventions based on PAR2 [57, 63, 67]. Strategies have used a mix of methods, ranging from in vitro cell culture studies, including human pancreas islet-derived precursor cells [54] and adipocyte-derived stem cell lines [60, 61] to gene transcription association studies in humans [62]. No evidence has been provided to indicate that regulation of endothelial cell-specific expression or activity contributes directly to the pathology of obesity, diabetes, or metabolic syndrome.

In regards to obesity, diabetes, and metabolic syndrome, there has been speculation that dipeptidyl peptidase-4 (DDP-4) [65] and chitinase-3 like protein 1 (CH3L1) [64] activate PAR2. However, weaknesses identified in Table 2 raise serious doubts about either of these proteins modulating specifically PAR2. More broadly the literature supports the notion that activation of PAR2 is not mediated by a single proteinase but is dependent on the protease network of local environments. It is unclear how exposure to proteases from food plants [58] fits into PAR2 regulation of metabolism or cardiovascular function. In theory, specific proteases could mediate different functions based on the tissue examined or its health state. In separate studies, some kallikreins were shown to activate PAR2 [80] and the kallikrein-kinin system is associated with the pathophysiology of diabetes, mostly by mechanisms involving bradykinin (reviewed in [81]). Cultured aortic vascular smooth muscle cells, but not endothelial cells, have been shown to activate plasma prekallikrein and stimulate PAR2 and epidermal growth factor receptor transactivation [82]. There is a current gap in direct evidence linking PAR2 to the kallikrein-kinin system in the pathophysiology of diabetes. However, the expression of PAR2 in tissues and organs (kidneys) susceptible to damage in diabetic humans [66] strengthens the rationale for further studies.

There have been few investigations conducted in humans that have found a relationship between PAR2 and obesity. A screening of thousands of single nucleotide polymorphisms in African Americans found a positive correlation between body mass index (BMI) and a PAR2 polymorphism [62]. In a separate small study, PAR2 mRNA expression in adipocyte tissues correlated with increasing the BMI of volunteer people [63]. Replication of these findings in other populations and identifying mechanisms underlying the single nucleotide polymorphism and activating agents would provide better understanding of scope of the impact on obesity and the significance if any for development of therapeutics targeting PAR2.

PAR2 is novel with regards to its response to vascular disease and is a pharmaceutical drug target. Vascular dysregulation is a common consequence of metabolic diseases, and new treatments are a priority for development. However, our understandings about PAR2 mechanisms in diabetes, obesity, and metabolic syndrome are based on preclinical laboratory models, which have included both type 1 and type 2 diabetic models. A point of controversy is whether PAR2 activation promotes obesity and, thus, further dysmetabolism in humans, because its expression correlated with BMI and at least in animal models, blocking PAR2 activity reduced adiposity and weight gain [57, 63]. Investigations conducted in humans indicate that PAR2-activating peptides increase forearm blood flow by arterial and venous dilation and were well-tolerated by volunteers [83, 84]. The discovery and development of new molecules to target PAR2 have been accelerating [24]. As newer agonist and antagonists of PAR2 [31, 34, 85] become available to researchers, the clinical potential of such compounds will become more clear.

In preparation of this review, we strived to comment on all published peer-reviewed articles relating to PAR2 in obesity, diabetes, and metabolic syndrome. The authors acknowledge that these metabolic conditions and their complications are complex and involve many physiological systems. By focusing on the direct vascular actions of PAR2 and the implications for cardiovascular complications, we have not commented on the potential contributions of PAR2 to other related complications through other mechanisms and systems, for example, neuropathy and pain [86], itch [87], and dermatitis [88]. This review is intended to provide interested researchers with a summary of the models and experimental approaches. In an attempt to summarize diverse studies, some details that make each piece of work unique have been omitted. However, there are editorials and reviews focused on specific articles cited in the review, for example, [5, 89], which can offer interested readers alternative insights.

## 5. Conclusions

PAR2 activation can produce endothelial cell specific regulation of vascular function in the face of obesity, diabetes, and metabolic syndrome. There is yet no evidence directly linking endothelial cell-specific PAR2 to obesity, diabetes, or metabolic syndrome. However, PAR2 expression is observed within other target tissues that could regulate vascular function, for example, perivascular adipose tissue. In general the role of PAR2 in producing obesity, diabetes, and metabolic syndrome has been investigated entirely independent of the function of the vascular system and supports the development of pharmaceutical antagonists of PAR2. However, further research is needed to investigate the mechanisms underlying PAR2 function in the vasculature and interaction with pathophysiology in metabolic diseases, especially the role of endothelial PAR2, which may be able to rescue vascular health.

## Figures and Tables

**Table 1:** PAR2 in obesity, diabetes, and metabolic syndrome: blood vessel function studies.

Model	Species	Sex	Strain	Age (weeks)	Metabolic phenotype			Vessel	PAR2 effects on blood vessels with endothelial dysfunction	PAR reagents	Article	Notes
					Glucose	Insulin	Body mass					
Nonobese diabetic (type 1 diabetes)	Mouse	F	NOD	5 13 22	Low High Severe	nd	Similar to control	Aorta	PAR2 dilation preserved by endothelial cell-independent mechanism. PAR2 de novo induction in vascular smooth muscle cells. COX-1 activity increased in vascular smooth muscle cells	PAR2-AP: SLIGRL; control AP: LSIGRL	[46]	Mean ages are listed; NOD/L_tj_ mice separated into groups by urinary glucose levels: low (0–20 mg/dL), high (20–500 mg/dL), severe 500–1000 mg/dL; CD-1 mice used as age-matched controls for body weight, data were not shown

Nonobese diabetic (type 2 diabetes)	Rat	M	Goto-Kakizaki	32–40	2.5 times control	1.6 times control	0.75 times control	Superior-mesenteric artery	PAR2 vasodilation sensitivity increased/preserved by endothelial cell PAR2-nitric oxide pathway	PAR2-AP: SLIGRL; control AP: LSIGRL	[47]	Control (Wistar age-matched) mean values for blood glucose, 170 mg/dL; plasma insulin, 2.8 ng/mL

Obese diabetic (type 2 diabetes)	Mouse	M	TallyHo	14–18	2.7 times control	Not different than control	Not different than control	Aortas with perivascular adipose tissue	Adipocyte-derived relaxing factor released by activation of PAR2	PAR2AP: SLIGRL, 2fLIGRLO; control AP: LRGILS, LSIGRL, 2fOLRGIL	[48]	Male TallyHo mice were reported as being hyperglycemic by Li et al. [48], but glucose, insulin, and body mass data were not shown. Phenotype data in Table 1 are from a separate study led by the same investigators [49]. Lean control (C57B6) and PAR2 knockout mice were age-matched to TallyHo mice. Control [49] (16 weeks of age) mean values: nonfasting blood glucose, 12.2 mM; serum insulin 0.8 *µ*g/g; body mass, 33 g. RT-PCR data provided evidence of PAR2 mRNA in adipocytes dissociated by collagenase treatment from perivascular aorta and mesenteric adipose tissue

Obese diabetic (type 2 diabetes)	Mouse	M	db/db	12–16	3 times control	nd	1.7 times control	Coronary microvessels	PAR2-AP vasodilation in vitro increased PAR2 antagonist treatment in vivo reduced endothelial dysfunction	PAR2-AP: 2fLIGRLO; control AP: 2fOLRGIL; PAR2 “putative” antagonist (in vivo) FSLLRY	[50]	Control (C57BL6) mean values for blood glucose, 156 mg/dL; body weight, 27 g. TNF knockdown mice crossed with db/db mice also were tested; no effect of FSLLRY-amide peptide on metabolic phenotype. Experiments were conducted using pressurized blood vessel assay

Obese diabetic (type 2 diabetes)	Mouse	M	db/db	12	2 times control	30 times control	2 times control	Second-order mesenteric artery	PAR2 vasodilation preserved by endothelium-dependent hyperpolarization factor	PAR2-AP: 2fLIGRLO; trypsin; control AP: 2fOLRGIL	[51]	Control (C57BL/6 age-matched) mean values for blood glucose, 11 mM; urinary glucose in db/db > 55 mM versus below assay detection limit (2 mM); serum insulin, 1.13 ng/mL; body mass, 27.5 g

Metabolic syndrome	Rat	M	SHRSP.Z-*Lepr* ^*fa*^.lz mDmcr (MetS)	13, 16, 23	1.7 times control	12.5 times control	1.2 times control	Superior and first-order branch mesenteric arteries	Preserved PAR2-mediated dilation by increased NO synthase contribution in MetS	PAR2-AP: 2fLIGRLO; control AP: 2fOLRGIL	[52]	Control (Wistar) mean values at 18 weeks of age: urine glucose, 187 mg/100 mL; serum insulin, 3.11 ng/mL; weights, 405 g; serum triglycerides, 46 mg/100 mL. MetS rats showed intolerance to elevation of blood glucose after oral glucose challenge. Systolic blood pressures (tail-cuff method) in MetS group were 60% higher than Wistar (110 mmHg) and decreased by administering in vivo telmisartan

High fat diet (HFD)	Rat	M	Sprague-Dawley	22–27	1.2 times control	nd	1.3 times control	Middle cerebral artery	Preserved EC-dependent PAR2 dilation by vascular smooth muscle large-conductance K^+^ channel mechanism Pressurized vessel assay	PAR2-AP: SLIGRL	[53]	Control (lean age-matched chow) mean values at nonfasting plasma glucose, 10 mM; body weights, 590 g. HFD (16–20 weeks treatment period) contained 2 times caloric content of chow diet. Retroperitoneal fat mass increased by 2.9-times (1.4% of body mass in control)

nd: variables were not determined; PAR2 activating and control peptides are identified by their amino acid sequences using the standard capitalized one-letter abbreviations. All peptides were synthesized as amides. Sequences starting with 2f indicate N-terminal modification with a 2-furoyl functional group.

**Table 2:** PAR2 in obesity, diabetes, and metabolic syndrome: nonblood vessel function studies.

Model	Species	Sex	Tissues	Age	Metabolic phenotype			PAR2 tissue/cell target	Mechanism insights	PAR reagents	Article	Notes
					Glucose	Insulin	Body mass					
Endocrine pancreas	Human	nr	Pancreas islets	—	—	—	—	Human islet-derived precursor cells (hIPC)	PAR2 is expressed in hIPCs; trypsin and PAR2-AP promote aggregation of hIPC which differentiate into islet-like aggregates	Trypsin; PAR2-AP: SLIGRL	[54]	Article identified while hand-searching the literature references

Insulin-deficient (type 1 diabetes)	Mouse C57B6	nr	Multiple	6–8 weeks	>3 g/dL urine	nr	nr	Mouse paw edema	Insulin signaling pathways may counter PAR2 mediated proinflammatory signaling in diabetic mice	PAR2-AP: SLIGRL; control AP: LRGILS	[55]	Diabetes is induced by T cell (effector Tc 1 cells with markers for CD8+ C3+ V*β*8.2+) mediated *β*-cell destruction. Metabolic phenotype monitoring was based on glycosuria > 3 g/dL. In-vitro data provided evidence that insulin reduced PAR2 stimulation of leukocyte adhesion to venular endothelium and global calcium influx in cell monolayers.

Obesity and diabetes	Mouse; *β*-arrestin 2 KO	M	Epididymal fat; liver	12–16 weeks	—	—	—	NIH3T3 fibroblasts; primary adipocytes; primary liver cells	PAR2 activation of Ca^2+^ signals increases cell metabolism by AMPK via CAMKK*β*; PAR2 activation of *β*-arrestins inhibits AMPK	PAR2-AP: 2fLIGRLO; control AP: 2fLRGLO	[56]	PAR2 activation of AMPK occurred only in cells isolated from *β*-arrestin knockout (KO) mice. Authors proposed that changes in *β*-arrestin in db/db mice could contribute to changes in PAR2 signaling and, thus, alter AMPK activity. Changes in the ratio of phospho-Thr^172^-AMPK to total AMPK protein and the phosphorylation of AMPK substrate ACC were used to assay AMPK activity

Obesity high fat diet (HFD)	Mouse; PAR2 KO	M	Adipocytes and adipose macrophages	22–24 weeks	75% of control	33% of control	88% of control	Multiple	Distinct cell-specific TF-PAR2 signalling promotes insulin resistance and obesity	Tissue factor (TF)	[57]	Starting at 6–8 weeks of age, PAR2 knockout (KO) and wild-type (WT) were fed a HFD, which provided 60% of their caloric intake from fat. Controls (WT HFD at 22–24 weeks of age) mean estimates from graphs for fasting plasma glucose, 250 mg/dL; fasting plasma insulin, 3 ng/mL; body mass, ~48 g. After HFD, plasma free fatty acid concentrations were lower, and the responses to challenges with glucose and insulin were better in PAR2KO than in WT. Whole-body energy expenditure, normalized to account for body mass differences, was 15% higher in HFD PAR2KO than in HFD WT

Diabetic (type 1 diabetes)	Mouse ICR	M	Ileum	nr	>300 mg/dL	nd	nd	Ileum	Bromelain (a cysteine protease) reduces the hypermotility of ileum in diabetic mouse. Preliminary evidence indicating inhibition of bromelain's in vitro effects by ENMD-1068	Bromelain; PAR2 antagonist: ENMD-1068; peptide: PAR2^C22-K36^	[58]	The effects of ENMD-1068 on the metabolic phenotype of the mice were not determined. Study provides evidence extending the concept of PAR2 regulation by proteinases like elastase [23] disarming the activating tethered ligand sequence. ENMD-1068 has been shown to be a low potency inhibitor of enzyme-mediated but not small peptide-mediated activation of PAR2 [39]

Obese diabetic (type 2 diabetes)	Mouse db/db	nr	Kidney	9, 20, and 30 weeks	2-3 times control	nd	1.8 times control	Kidney tissues	Three-times at 20 weeks, and two times at 30 weeks more PAR2 positive-stained cells in glomerulus of db/db	none	[59]	Control (9 weeks of age db/db) group mean values: blood glucose, 429 mg/dL. Immunohistochemistry methods identified PAR2-specific staining in glomerulus of kidney tissue sections. Similarly, collagen and fibrin deposition increased in the same sections with age of db/db and reduction in kidney function

Adipose	Human	M F	Adipose-derived stem cell lines	42–58 years	—	—	—	—	PAR2-specific immunofluorescence identified on plasma membrane of adipose-derived stem cells in culture. Data obtained with trypsin alone and in combination with blocking antibody provides evidence suggesting a role for PAR2 mediating an upregulation of VEGF expression in these cells	Trypsin, antibody (SAM-11)	[60]	Cell lines were derived from 3 subjects [61], which were described as being without cardiovascular disease

Obesity	Human	M F	—	—	—	—	BMI >30 kg/m^2^	—	F2Rl1 on Chr 5 associated with BMI	none	[62]	Data collected from 1733 unrelated African Americans were combined in an admixture mapping study; F2Rl1 correlations with hypertension, systolic arterial blood pressures, diastolic arterial blood pressures, and high density lipoprotein C were not significant

Obesity	Human	nr	Omental and subcutaneous fat	—	—	—	BMI (kg/m^2^): lean, 21.4; overweight, 27.3; obese, 32.9.	Monocyte derived macrophages	Evidence indicated a positive linear correlation between increasing body-mass index and the levels of PAR2 mRNA expression in omental fat (*n* = 11 subjects); a twofold difference in PAR2 mRNA expression was seen over the range of BMI tested. Palmitic acid induced PAR2 expression in cultured monocyte-derived macrophages	PAR2 antagonist: GB88; PAR2AP: SLIGRL, 2fLIGRLO; antibodies: N19, SAM11	[63]	Rats (8-9 weeks of age) were fed a high-carbohydrate high-fat (HCHF) diet for a 16-week period. From weeks 8 to 16, rats were treated daily with either control (olive oil) or PAR2 antagonist GB88 (10 mg/kg/day). Control group (HCHF) means estimated from graphs for body mass at 24-25 weeks of age, 530 g; cumulative weight gain from weeks 8 to 16, 20%. GB88-treated rats showed better in vivo responses to challenges with glucose and insulin
Obesity/MetS treatment	Wistar rats	M	In vivo	24-25	—	—	Cumulative weight gain 50% of controls	Multiple nonvascular cells	Inhibition in vivo of PAR2 slowed weight gain, which seemed to be due decrease in the mass of fat accumulated during the period of the diet-induced obesity. Other beneficial effects of GB88 included normalizing plasma lipids, cholesterol, liver injury, and promoting metabolism gene expression in skeletal and adipose tissue			

Insulin resistance	Human	M F	Skeletal myotubes and myoblasts	16–47 years	—	—	—	—	CHI3L1 protected cultured skeletal myoblasts from TNF-alpha induced insulin resistance. Evidence suggested CHI3L1 inhibition of TNF-alpha activation of NFkB was PAR2-dependent	CH3L1; trypsin; PAR2AP (SLIGKV); PAR2 inhibitory antibody: SAM11	[64]	The mechanism by which Chitinase-3 like protein 1 (CH3L1) interacts with PAR2 is not yet determined. PAR2 mRNA and protein expression were identified in the cell preparations; however, this study stopped short of directly testing known PAR2 activators in the cell culture model of insulin resistance. Alternate names for CH3L1 include mouse BRp39 (breast regression protein 39) and YKL-40

Metabolic syndrome	Human	nr	Coronary artery smooth muscle cells	—	—	—	—	Cultured cells	sDDP4 is a candidate adipokine, which may cause proliferation of coronary artery smooth muscle cells via PAR2	sDDP4; PAR2AP: SLIGKV; PAR2 antagonist: GB83	[65]	The mechanism by which soluble dipeptidyl peptidase 4 (sDDP4) activates PAR2 remains unclear. The authors speculated about nature of a four-amino-acid length conserved sequence between the human tethered ligand of PAR2 and sDDP4. Studies cited by the authors provide correlative evidence between sDDP4 expression and metabolic syndrome parameters

Diabetic nephropathy (DN)	Human	M F	Kidney	47–65 years (DN)	nr	nd	nr	Cortical sections	Increased immunohistochemistry staining for PAR2 in DN (2-3 times control), which was higher in vascular and tubular cells than in glomeruli	PAR2 antibody	[66]	Archival renal biopsy samples from 5 DN and 5 controls (normal portions from renal carcinoma cases). Average period of DN was 6.2 years at time of biopsy

Obesity (HFD)	Mouse tissue factor cytoplasmic tail deficient knock-in (TF-KI)	M	Liver	25–27 weeks	80% of controls	75% of controls	Same as controls	Liver and hematopoietic cells	TF-PAR2 signalling pathways in hepatocytes and hematopoietic cells independently contribute to steatosis	Antibody: 10H10 antibody	[67]	TF-KI were fed HFD for 19 weeks. From weeks 16–19, mice were administered 10H10 antibody or IgG (controls). Metabolic phenotype variables (means) are estimated from graph data in [67]. Control group: plasma glucose, 230 mg/dL; fasting plasma insulin, 5 ng/mL; body mass reported the same as wild-type mice, but data were not provided. PAR2 mRNA expression was 5 times higher in livers from HFD versus controls

nr, data values were not reported; nd, not determined; —, category is not directly applicable to study.

## Abbreviations

BMI: Body mass index
CH3L1: Chitinase-3 like protein 1
DPP-4: Dipeptidyl peptidase-4
GPCR: Heteromeric G protein-coupled receptors
HCHF: High-carbohydrate high-fat
PAR2: Proteinase-activated receptor 2; protease-activated receptor 2; factor 2-receptor like 1; GPCR 11.
